# Streamflow contributions from tribal lands to major river basins of the United States

**DOI:** 10.1371/journal.pone.0203872

**Published:** 2018-09-11

**Authors:** Kyle Blasch, Stephen Hundt, Patrick Wurster, Roy Sando, Antony Berthelote

**Affiliations:** 1 Idaho Water Science Center, US Geological Survey, Boise, Idaho, United States of America; 2 Natural Resources Program, Salish Kootenai College, Pablo, Montana, United States of America; 3 Wyoming-Montana Water Science Center, US Geological Survey, Helena, Montana, United States of America; Universiti Sains Malaysia, MALAYSIA

## Abstract

While many studies on tribal water resources of individual tribal lands in the United States (US) have been conducted, the importance of tribal water resources at a national scale has largely gone unrecognized because their combined totals have not been quantified. Thus, we sought to provide a numerical estimate of major water budget components on tribal lands within the conterminous US and on USGS hydrologic unit codes (HUC2) regions. Using existing national-scale data and models, we estimated mean annual precipitation, evapotranspiration, excess precipitation, streamflow, and water use for the period 1971–2000. Tribal lands represent about 3.4 percent of the total land area of the conterminous US and on average account for 1.9 percent of precipitation, 2.4 percent of actual evapotranspiration, 0.95 percent of excess precipitation, 1.6 percent of water use, and 0.43 percent of streamflow origination. Additionally, approximately 9.5 and 11.3 percent of US streamflow flows through or adjacent as boundaries to tribal lands, respectively. Streamflow through or adjacent to tribal lands accounts for 42 and 48 percent of streamflow in the Missouri region, respectively; and for 86 and 88 percent in the Lower Colorado region, respectively. On average, 5,600 million cubic meters of streamflow per year was produced on tribal lands in the Pacific Northwest region, nearly five times greater than tribal lands in any other region. Tribal lands in the Great Lakes, Missouri, Arkansas-White-Red, and California regions all produced between 1,000 and 1,400 million cubic meters per year.

## Introduction

**Fig 1:**
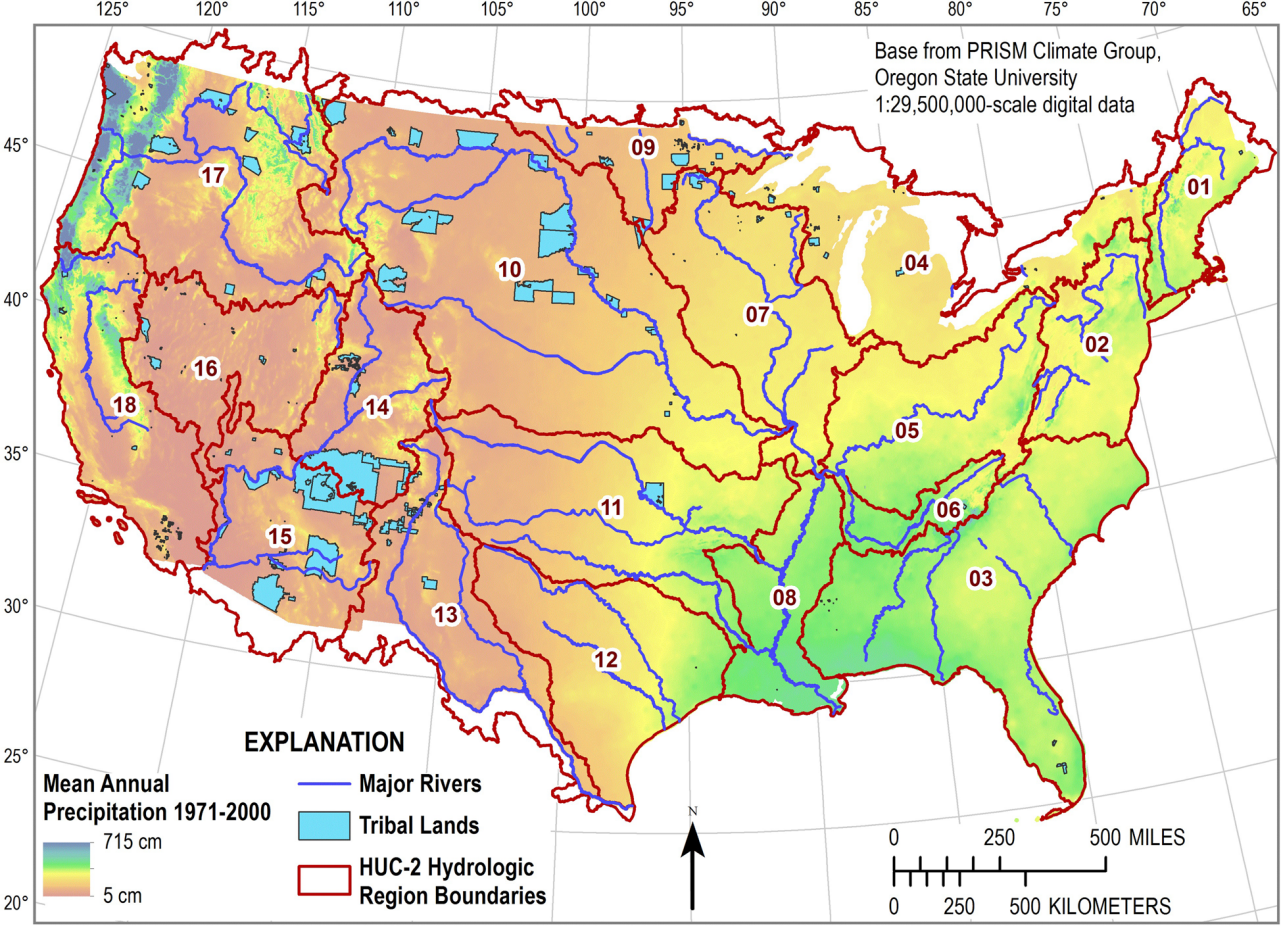
Map of the conterminous United States with federally-recognized tribal lands, 2-digit hydrologic unit code regions, major rivers, and precipitation data for 1971–2000 [30].

Surface water has historically been essential to the establishment and survival of tribal communities within the conterminous US [1, 2] and continues to be important to the sustainability and success of these communities [3–6].The US Bureau of Indian Affairs currently (US Federal Register January 17, 2017) recognizes 567 federally-recognized tribes spread across the US (339 in the conterminous US) with the largest tribal lands, by land area, located in the western half of the US (Fig 1). Approximately 1.14 million American Indians and Alaska Natives permanently reside on US tribal lands [4].

Studies on the water resources of individual tribal lands have been conducted for a diverse set of reasons which include quantifying water availability [7–11], adjudicating water rights [1], estimating sedimentation rates [12, 13], defining water quality issues [14, 15], supporting agriculture [16], managing fisheries [17, 18], estimating recharge of groundwater systems [19], managing sea water intrusion [20], water resources planning [3, 6], ecosystem health [21], responding to climate change [22, 23], and managing resources to preserve Native American traditional practices [24, 25]. These local studies describe not only the unique aspects of tribal water resources but also the interrelationships between land-use practices and water resources management occurring on and adjacent to tribal lands.

Despite the number of local and disparate efforts to study water resources on tribal lands, the importance of tribal water resources at a national scale has largely gone unrecognized due to a lack of quantification of the collective resource. The advancement of national datasets describing water budget components, such as precipitation and streamflow, offers the ability to estimate these quantities with reasonable accuracy in relation to the basins in which tribal lands exist. The conterminous US is spatially divided into 18 major hydrologic regions defined by USGS hydrologic unit codes (HUC2) representing the major rivers of the US [26–28]. Each region contains one or more major US river basins. Rivers from one region may drain into a downstream region, as is the case for the Mississippi and Colorado Rivers. The objective of this investigation was to provide a numerical estimate of long-term mean water budget components, primarily streamflow, on tribal lands within the conterminous US at the national and HUC2 regional levels to better understand the relation between tribal and non-tribal lands for improved management of water resources.

## Methods

Water budgets were developed for tribal and nontribal lands at the national and HUC2 region levels for a comparison of hydrologic inputs and outputs. A generalized water balance compares inputs of precipitation, streamflow, and groundwater with the outputs of evapotranspiration, consumptive water use, streamflow, and groundwater. National datasets (1971–2000) are available for precipitation, evapotranspiration, streamflow, and water use and are discussed below. Precipitation is the major water input into the HUC2 regions with smaller contributions from streamflow transferred to the Lower Colorado and Lower Mississippi HUC2 regions from upstream regions. Other water inputs to HUC2 regions from groundwater and engineered transfers of water may also occur between regions but were not considered in this analysis. The water budgets could not be fully closed as groundwater terms could not be found at national and HUC2 levels. Yet comparison of each input and output value between the tribal lands and the accompanying HUC2 regions was still informative.

### Tribal lands and watershed boundaries

Tribal land boundaries and areas were represented using the National Atlas of the United States [29]. This dataset includes the political boundaries of tribal lands within the conterminous US that are larger than 259 hectares (640 acres) in size and administered by the US Bureau of Indian Affairs. Tribal lands may include reservations, pueblos, Indian communities, rancherias, off-reservation trust lands and other administered lands. The boundaries of the tribal lands from this dataset were used to extract data from the geospatial datasets used in this investigation. Areas within HUC2 regions extending beyond US borders into Canada and Mexico were not considered in this analysis.

### Precipitation

The simplest means to quantify the contribution of surface water on tribal lands to national and regional HUC2 water resources is through the quantification of precipitation on tribal and non-tribal lands. Comparatively, anthropogenic influences and management affect precipitation inputs less than other water budget components. Once precipitated, rain and snowmelt can infiltrate into groundwater systems, run off, be used for irrigation or another consumptive use, or return to the atmosphere through evapotranspiration or sublimation.

Mean annual precipitation was calculated using the PRISM (Parameter elevation Regression on Independent Slopes Model) dataset for 1971–2000 [30]. The period 1971–2000 was selected because it was the only multi-decadal time period when precipitation, evapotranspiration, and streamflow datasets were available. The PRISM dataset was selected because it is generated for the entire conterminous United States, incorporates observed precipitation data, and incorporates weighted regressions to account for variable geography [27, 31]. The uncertainty of the annual PRISM data was estimated using single-deletion jackknife cross validation and PRISM 70% prediction interval and was on average about four percent in the Eastern U.S, five percent in the Central U.S. and about 11 percent in the West (Daly, Halbleib et al. 2008).

### Actual evapotranspiration and excess precipitation

The mean annual actual evapotranspiration (AET) data were extracted from a national dataset produced by Sanford and Selnick (2013; 32) derived from regressions based on 838 nationwide streamflow gaging stations, PRISM climate data sets, and USGS national land cover datasets for the period 1971 to 2000 [32]. The stations were selected based on period of record and minimal upstream streamflow regulation or withdrawals. AET is a more appropriate water budget component than potential ET as it is a measure of the water that actually evapotranspires from the landscape based on available water and prevailing climate conditions. Potential ET is the maximum amount of water that could evaporate or transpire from the land surface to the atmosphere if the water was available. Often in arid climates AET is a smaller quantity than PET because there is no available water. The AET estimates were derived using climate and land cover factors but did not consider other withdrawals which may influence the values. The uncertainty for the AET dataset is on average about 6.6 percent of HUC2 regional dataset [32].

Mean annual excess precipitation was calculated as mean annual precipitation minus mean annual AET and is an estimate of precipitation remaining for infiltration or runoff after AET is removed. Ignoring the groundwater contribution to streamflow, excess precipitation is an upper limit of water available for contribution to streamflows from tribal lands at the conterminous US and HUC2 regional scales. While groundwater is an important source of water to streams as baseflow, it is primarily supported by recharge from excess precipitation.

### Streamflow

Streamflow originating on tribal lands was calculated using National Hydrography Dataset Plus Version 2 (NHDplusV2) surface water discharge values [33]. The NHDplusV2 is an open source geospatial dataset developed by the Environmental Protection Agency (EPA) and U.S. Geological Survey (USGS) and provides comprehensive information describing surface water characteristics within the US [34]. Flowlines and corresponding surface water discharge estimates based on the enhanced runoff method (EROM) were of particular importance. Flowlines are digital representations of flowing surface water and either have known or unknown flow direction. Flowlines with known (“with digitized”) flow direction contained stream order, EROM produced streamflows, and other important catchment information. Flowlines with unknown (“uninitialized”) flow direction did not contain these data and largely consisted of irrigation canals, isolated streams, or braided stream segments [34]. The EROM streamflow values were calculated using a water balance model calibrated to data collected at USGS streamflow-gaging stations with a minimum period of record of 20 years between 1971 and 2000 [34]. The NHDPlusV2 User Guide identifies the EROM Q0001E field as containing the most accurate streamflow discharge estimates for each flowline segment [34]. Furthermore, the uncertainty with this dataset can be obtained with the download of this dataset and varies depending on location [34].

**Fig 2:**
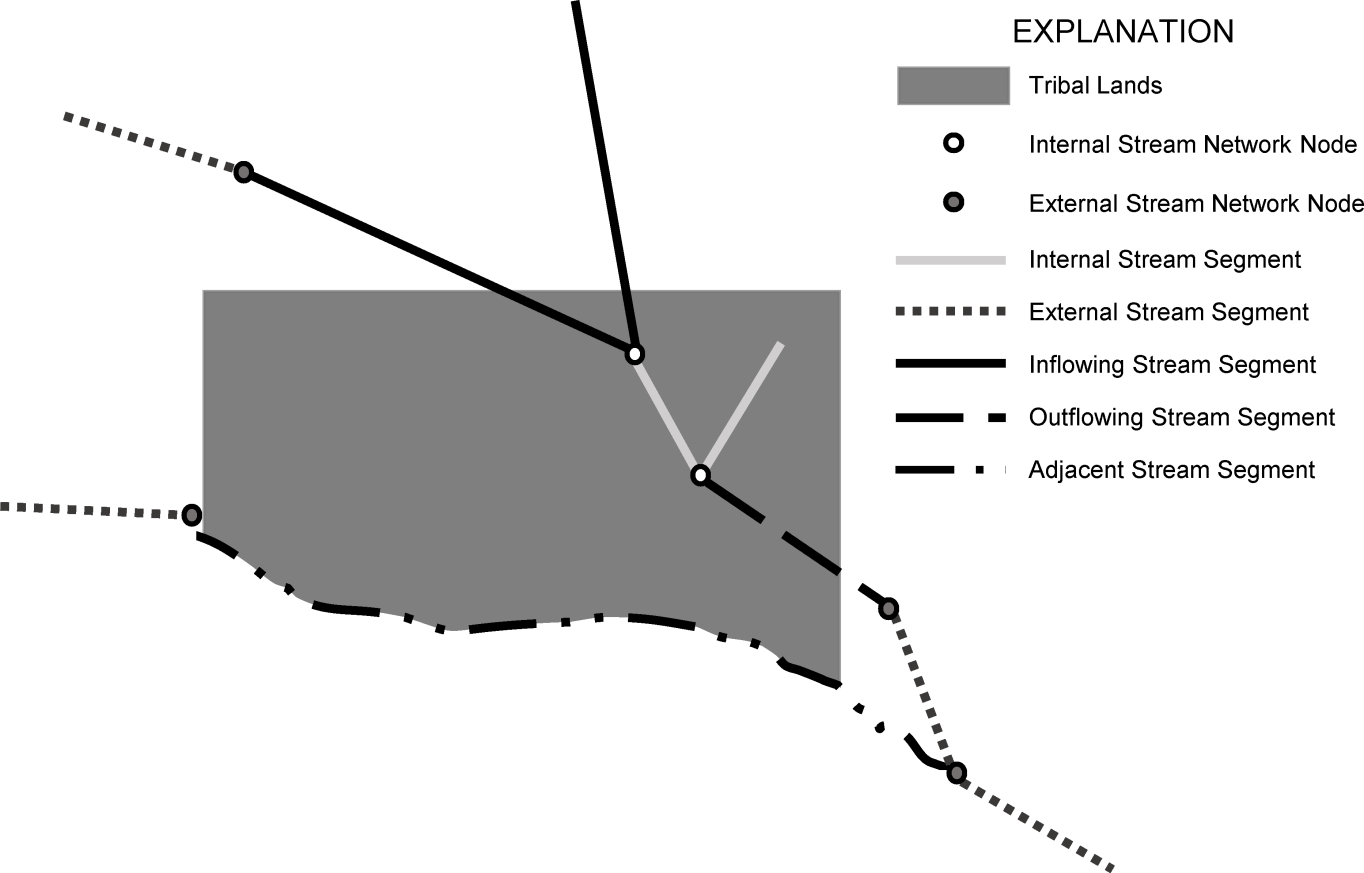
Method for categorizing stream segments near tribal lands. Individual stream segments join at nodes, which are internal (white) or external (gray) to a tribal land. Five stream segment types are defined by type of nodes that form their endpoints: inflowing segments (black solid line) flow from an external to an internal node, outflowing segments (black dashed line) flow from internal to external, interior segments (white solid line) flow from internal to internal, and exterior segments (gray dotted line) flow from external to external. Finally, segments that flow adjacent to a tribal land boundary (black dashed-dotted lines) were defined manually.

Streamflow values were obtained by identifying where flowlines intersected tribal lands and HUC2 region boundaries and obtaining the discharge estimate for the flowline segment (Fig 2). Streamflow gains for each tribal land polygon were calculated as the total streamflow entering the polygon subtracted from the streamflow exiting the polygon. This was a negative value in some cases, which indicated withdrawals for water use, natural water losses such as infiltration through the streambed, and uncertainty with the datasets. Streams and rivers often run adjacent to different tribal lands which was the impetus for additionally quantifying adjacent waters (Fig 2). Streamflows that flowed through or adjacent to multiple tribal lands were only considered once for the tribal lands water budgets. Using a manual procedure, only the largest (most downstream) streamflow values along a stream course adjacent to or flowing through different tribal lands were used in the computation to avoid double counting these streamflows. This correction was applied only for the main stem of major rivers and not in cases where small tributary streams pass through one tribal land and join the main stem before they pass through or along another tribal land.

### Water use

Mean consumptive water use data were compiled using USGS data from 1980 through 1995 [35–37]. Unfortunately, water use datasets are not available to span the full time period from 1971–2000 but the 15 years of data provide an estimate of water use during this period. Fresh surface water use and fresh groundwater use were summed for each HUC2 region using the values for the smaller HUC8 units reported in the USGS compilations. For the tribal land polygons, the tribal boundaries and the HUC8 boundaries were overlain and water use values were weighted proportionally to the difference in land area where the boundaries of the tribal lands and the HUC8 regions were not aligned. Water use data were not used to adjust streamflow values as there were insufficient data description and resolution to associate the use with particular streams and discharge points. Uncertainty estimates were not provided with these datasets, which are the best available.

## Results

### Land area

**Fig 3:**
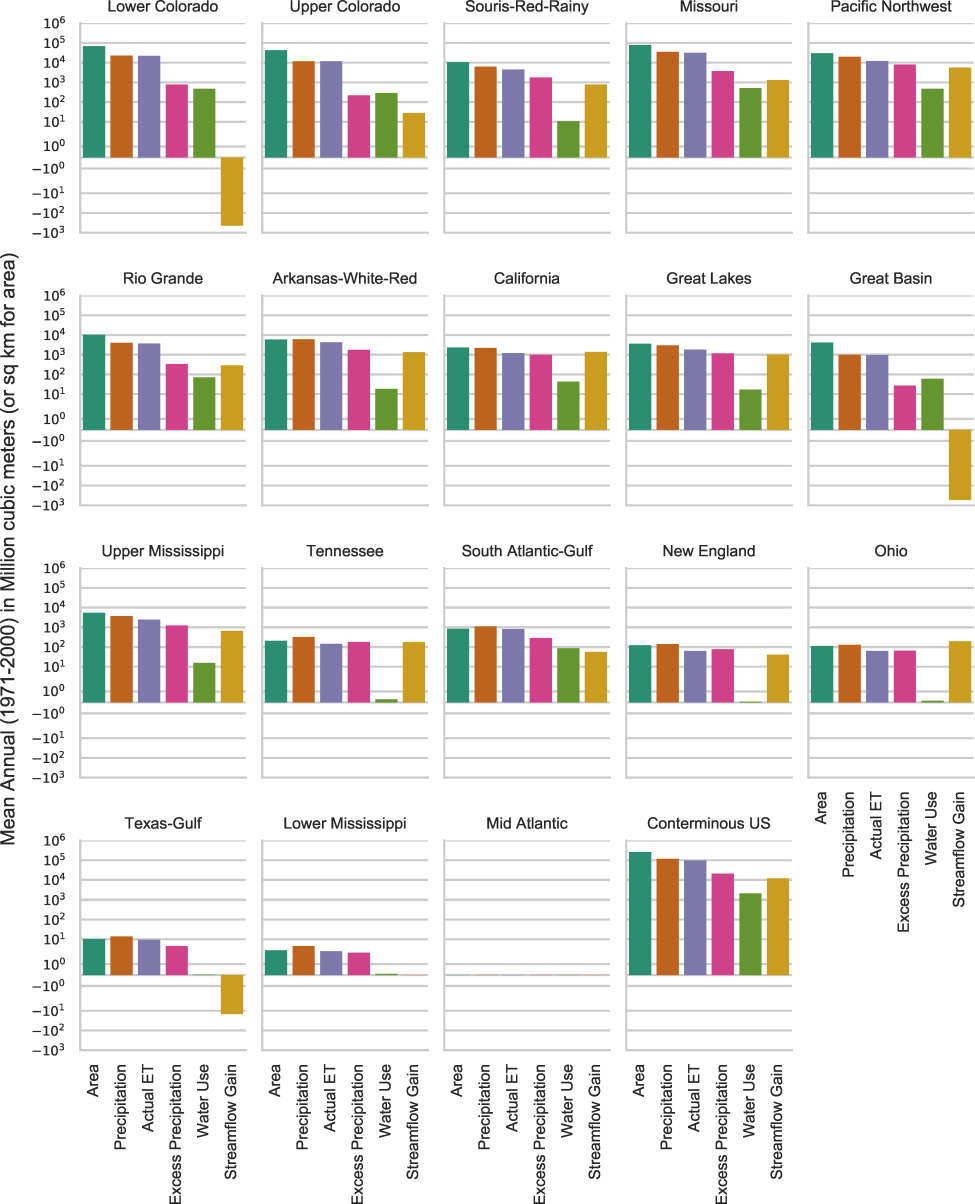
Tribal water budget and areal estimates for the conterminous United States and HUC2 regions. See Tables 1 and 2 for values. Note that the y-axis is a logarithmic scale to enable comparison of all values.

**Fig 4:**
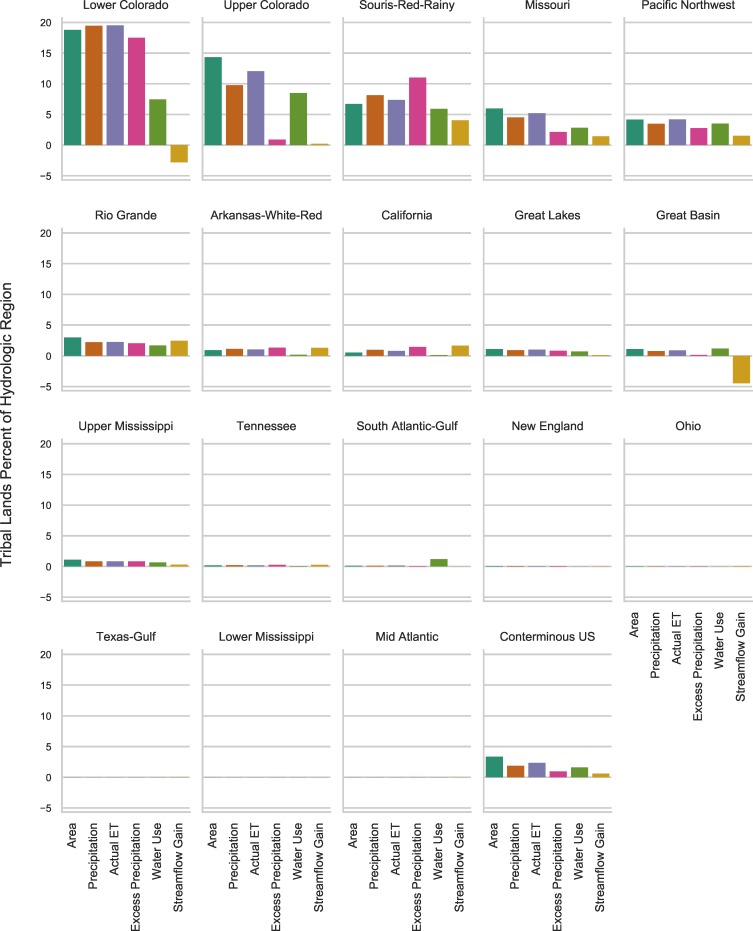
Tribal water budget and areal estimates as a percentage of conterminous United States and HUC2 regions. See Tables 1 and 2 for values. Note that the y-axis is a linear scale.

The total land areas for the conterminous US and tribal lands are approximately 7,787,300 square kilometers (km^2^) and 261,800 km^2^, respectively (Fig 3 and Table 1). Tribal lands account for about 3.36 percent of the conterminous US, with the western HUC2 regions containing larger percentages of tribal lands (Fig 4 and Table 1). Tribal lands in the Lower and Upper Colorado basins account for the largest portion of their respective HUC2 regions, at 18.8 and 14.3 percent, respectively. The Mid Atlantic HUC2 region does not contain any tribal lands. All water balance results for this investigation can be downloaded from ScienceBase at https://doi.org/10.5066/F7959GR6 [38].

**Table 1:** Water budget calculations for tribal lands within 2-digit hydrologic unit code regions.

**HUC2 Number**	**HUC2 Name**	**Area**	**Precipitation**	**AET**	**Excess Precipitation**	**Streamflow Origination**	**Water Use**
		**km**^**2**^	**MCM**	**MCM**	**MCM**	**MCM**	**MCM**
1	New England	122	139	63	76	41	0.06
2	Mid Atlantic		0	0	0	0	0
3	South Atlantic-Gulf	829	1,107	821	287	56	86.08
4	Great Lakes	3,606	2,959	1,804	1,154	1,013	16.97
5	Ohio	112	128	63	65	197	0.14
6	Tennessee	205	321	142	178	177	0.28
7	Upper Mississippi	5,441	3,671	2,454	1,218	653	15.62
8	Lower Mississippi	3	5	2	2	0	0.10
9	Souris-Red-Rainy	10,390	6,296	4,508	1,788	772	10.60
10	Missouri	78,763	34,785	31,102	3,683	1,287	507.90
11	Arkansas-White-Red	5,958	5,976	4,204	1,772	1,337	18.61
12	Texas-Gulf	10	14	10	5	-15	0.01
13	Rio Grande	10,206	4,008	3,666	342	286	70.34
14	Upper Colorado	42,074	11,805	11,583	222	28	289.98
15	Lower Colorado	68,114	22,642	21,864	778	-444	476.52
16	Great Basin	4,100	995	968	27	-543	59.62
17	Pacific Northwest	29,527	20,005	12,039	7,966	5,626	466.73
18	California	2,311	2,179	1,197	983	1,384	43.53
**TOTAL**	**All Tribal Lands**	**261,771**	**117,035**	**96,489**	**20,546**	**11,856**	**2063.09**
**HUC2 Number**	**HUC2 Name**	**Area**	**Precipitation**	**AET**	**Excess Precipitation**	**Streamflow Origination**	**Water Use**
		**% of HUC2**	**% of HUC2**	**% of HUC2**	**% of HUC2**	**% of HUC2**	**% of HUC2**
1	New England	0.079	0.077	0.081	0.074	0.04	0.0065
2	Mid Atlantic	0	0	0	0.000	0.00	0.0000
3	South Atlantic-Gulf	0.119	0.118	0.150	0.073	0.02	1.1808
4	Great Lakes	1.104	0.943	1.016	0.847	0.12	0.7201
5	Ohio	0.027	0.027	0.023	0.033	0.07	0.0054
6	Tennessee	0.194	0.220	0.182	0.264	0.28	0.0647
7	Upper Mississippi	1.106	0.838	0.844	0.827	0.31	0.6505
8	Lower Mississippi	0.001	0.001	0.001	0.001	0.00	0.0011
9	Souris-Red-Rainy	6.700	8.123	7.358	11.004	4.04	5.9245
10	Missouri	5.957	4.515	5.192	2.149	1.44	2.8251
11	Arkansas-White-Red	0.928	1.127	1.059	1.331	1.32	0.1800
12	Texas-Gulf	0.002	0.004	0.003	0.005	-0.03	0.0002
13	Rio Grande	2.974	2.204	2.220	2.052	2.45	1.7048
14	Upper Colorado	14.332	9.768	12.033	0.901	0.21	8.4995
15	Lower Colorado	18.765	19.453	19.530	17.505	-2.80	7.4511
16	Great Basin	1.117	0.792	0.896	0.154	-4.47	1.2102
17	Pacific Northwest	4.158	3.476	4.189	2.764	1.52	3.5029
18	California	0.556	1.000	0.796	1.450	1.66	0.1400
**TOTAL**	**All Tribal Lands**	**3.36**	**1.8719**	**2.3588**	**0.950**	**0.43**	**1.6011**

Area data were calculated using boundaries from the National Atlas of the United States (NAUS, 2014). Precipitation data were calculated using PRISM (Parameter elevation Regression on Independent Slopes Model (NCAR, 2015). Actual evapotranspiration (AET) data were extracted from a national dataset produced by Sanford and Selnick (2013). Streamflow originating on tribal lands was calculated using National Hydrography Dataset Plus Version 2 (NHDplusV2) surface water discharge EROM (enhanced runoff method) values (UESPA, 2012). Abbreviations: AET, actual evapotranspiration; HUC2, hydrologic unit code 2; km2, square kilometers; MCM, million cubic meters; %, percent; US, United States]

**Table 2:** Streamflow estimates of inflows, outflows, gains, and adjacent flows for tribal lands and 2-digit hydrologic unit code regions.

HUC2 Number	HUC2 Name	HUC2 Streamflow	Reservation inflows	Reservation outflows	Reservation outflows	Reservation streamflow origination	Reservation streamflow origination	Reservation streamflow adjacent	Reservation streamflow adjacent
		MCM	MCM	MCM	% of HUC2	MCM	% of HUC2	MCM	% of HUC2
1	New England	101,885	452	493	0.48	41	0.04	13,380	13.13
2	Mid Atlantic	132,150	0	0	0	0	0	0	0
3	South Atlantic-Gulf	282,896	1,059	1,115	0.39	56	0.02	0	0.00
4	Great Lakes	847,317	10,709	11,722	1.38	1,013	0.12	9,859	1.16
5	Ohio	274,593	2,759	2,956	1.08	197	0.07	0	0.00
6	Tennessee	63,526	2,067	2,245	3.53	177	0.28	0	0.00
7	Upper Mississippi	212,286	3,340	3,994	1.88	653	0.31	1,625	0.77
8	Lower Mississippi	860,804	0	0	0.00	0	0.00	0	0.00
9	Souris-Red-Rainy	19,106	3,115	3,887	20.35	772	4.04	120	0.63
10	Missouri	89,576	36,447	37,734	42.13	1,287	1.44	43,383	48.43
11	Arkansas-White-Red	101,267	986	2,323	2.29	1,337	1.32	7,480	7.39
12	Texas-Gulf	50,213	171	156	0.31	-15	0.00	0	0.00
13	Rio Grande	11,674	21,499	6,465	55.38	-15,034	2.45	2,030	17.39
14	Upper Colorado	13,425	15,463	15,490	115.39	28	0.21	13,163	98.05
15	Lower Colorado	15,819	20,910	13,623	86.12	-7,287	0.00	13,978	88.36
16	Great Basin	12,145	792	249	2.05	-543	0.00	82	0.68
17	Pacific Northwest	370,003	54,167	59,793	16.16	5,626	1.52	110,634	29.90
18	California	83,618	21,373	22,757	27.22	1,384	1.66	3,902	4.67
**TOTAL**		**1,940,312**	**195,310**	**185,002**	**9.53**	**11,856**	**0.43**	**219,637**	**11.32**

Streamflow originating on tribal lands was calculated using National Hydrography Dataset Plus Version 2 (NHDplusV2) surface water discharge EROM (enhanced runoff method) values (UESPA, 2012). Abbreviations: HUC2, hydrologic unit code 2; MCM, million cubic meters; %, percent;

### Precipitation

The mean annual precipitation from 1971 through 2000 for the conterminous US was estimated to be 6.25 trillion cubic meters (m^3^) or about 5.07 billion acre-feet (AF). The mean annual precipitation from 1971 through 2000 for the tribal lands in the conterminous US was about 117 billion m^3^ (94.9 million AF), which is about 1.87 percent of mean annual precipitation in the conterminous US (Figs 3 and 4). Precipitation, the water budget component least affected by anthropogenic effects, is the most suitable estimate of water resources contributions from tribal lands within the US. The precipitation contribution of 1.87 percent is smaller than the land area percentage because the largest tribal lands are in arid and semi-arid areas where precipitation rates are smaller compared to US average precipitation. Precipitation contributions within each HUC2 region vary considerably and are closely correlated with the relative size of tribal lands and geography (e.g., topography and climate) within each region. The Missouri region has the largest mean annual precipitation on tribal lands of 34.8 billion m^3^ (28.2 million AF), which only constitutes about 4.5 percent of precipitation in the HUC2 region. The Lower Colorado region tribal lands contribute 22.6 billion m^3^ (18.4 million AF), which is about 19.45 percent of precipitation in the HUC2 region.

### Actual evapotranspiration

Mean annual AET from 1971 through 2000 for the conterminous US was about 4.09 trillion m^3^ (3.32 billion AF). Mean annual AET from 1971 through 2000 for tribal lands in the conterminous US was approximately 96.5 billion m^3^ (78.2 million AF), about 2.36 percent of the total for the conterminous US (Figs 3 and 4). The tribal lands AET percentage is smaller than the tribal land area percentage because within arid and semi-arid areas water available for AET is more limited than in other parts of the US. The tribal lands AET percentage is larger than the tribal lands precipitation percentage because of the proportionately higher temperatures and lower humidity of the tribal lands compared to conterminous US national averages.

### Excess precipitation

Mean annual excess precipitation from 1971 through 2000 for the conterminous US was about 2.16 trillion m^3^ (1.75 billion AF). Mean annual excess precipitation from 1971 through 2000 for tribal lands in the conterminous US was 20.5 billion m^3^ (16.7 million AF), about 0.95 percent of the total for the conterminous US (Figs 3 and 4). Excess precipitation was 34.6 percent of precipitation for the conterminous US and was 17.6 percent of precipitation for all tribal lands. The smaller excess precipitation on tribal lands is attributed to less precipitation on these lands and larger AET compared to non-tribal lands. Among tribal lands, those in the Pacific Northwest HUC2 region contribute the most excess precipitation with 7.97 billion m^3^ (6.46 million AF) because of the large total area of tribal lands and high precipitation to AET ratios.

### Water use

Mean annual water use on tribal lands from 1980 to 1995 was 1.60 percent of all conterminous US water use. The relatively small water use percentage for tribal lands compared to the tribal lands area percentage (1.60% vs. 3.36%, respectively) may be attributed to the lower population density, less intensive agriculture, greater aridity of tribal lands and more limited infrastructure for domestic and agricultural uses compared to non-tribal lands [39]. Tribal water use ranged from 0.0002 to 8.5 percent of HUC2 regional water use, with a median value of 0.69 percent (Table 1). The Missouri and Lower Colorado regions have the largest areas of tribal lands and also the highest water use, at 508 and 477 million m^3^ per year, respectively (Table 1).

### Streamflow

The conterminous US had an estimated mean annual streamflow discharge across US conterminous borders of about 2.79 trillion m^3^ (2.26 billion AF) using NHDplusV2 surface water discharge values. It is important to note that streamflow estimates based on EROM calculations do not fully account for water use withdrawals and losses of streamflow through infiltration and evapotranspiration. Thus, relative comparisons of streamflow percentages between tribal lands and HUC2 regions are more reliable than the absolute quantities. Approximately 12.9 billion m^3^ (10.4 million AF) mean annual streamflow originated on tribal lands and accounted for about 0.43 percent of total streamflow exiting the conterminous US (Fig 4) which include streamflow contributions from the internal HUC2 regions. Tribal lands in the Pacific Northwest region generate the most streamflow of any region (5,626 million m^3^per year) due to the humid climatic conditions and large area of tribal lands (Fig 3). Tribal lands in the Great Lakes, Missouri, Arkansas-White-Red, and California HUC2 regions all produced mean annual streamflow between 1,000 and 1,400 million m^3^.

Overall, the mean annual volume of streamflow originating on tribal lands is less than the mean annual volume of excess precipitation, although a few exceptions exist. The Ohio and California regions have higher streamflow than excess precipitation values (Table 1). Both regions have tribal lands that contain long and narrow river valleys in humid areas with USGS streamflow gaging stations near the outflows from tribal lands. The NHDplus streamflow dataset showed an abrupt increase in discharge in the stream reaches containing USGS gaging stations in both regions. Thus, there is potentially a systematic underestimation of excess precipitation in the valley bottoms of both regions. Other explanations are an underestimation of inflows to tribal lands and main stem streams from ungaged tributaries, or unaccounted sources of streamflow, such as groundwater or interbasin transfers where the groundwater basin boundaries do not align with the surface watershed boundaries.

The Texas-Gulf, Lower Colorado, and Great Basin regions all have streamflow losses for their tribal lands (Table 1). This indicates that more streamflow enters than leaves the tribal lands. While some individual tribal lands produce streamflow gains, overall the streamflow losses are greater. Factors for the losses include arid climates, losing streams (streamflow infiltration through the bed sediments), and consumptive water use (e.g., irrigation). Water use is about 219 percent of excess precipitation for the Great Basin region. In the Texas-Gulf region, inflows to tribal lands of 171 million m^3^ nearly equal outflows of 156 million m^3^ and the difference is within the bounds of the accuracy of the EROM estimates (Table 2). Water use in the Texas-Gulf region is low and the net loss of streamflow may be attributed to the accuracy of the data.

**Fig 5:**
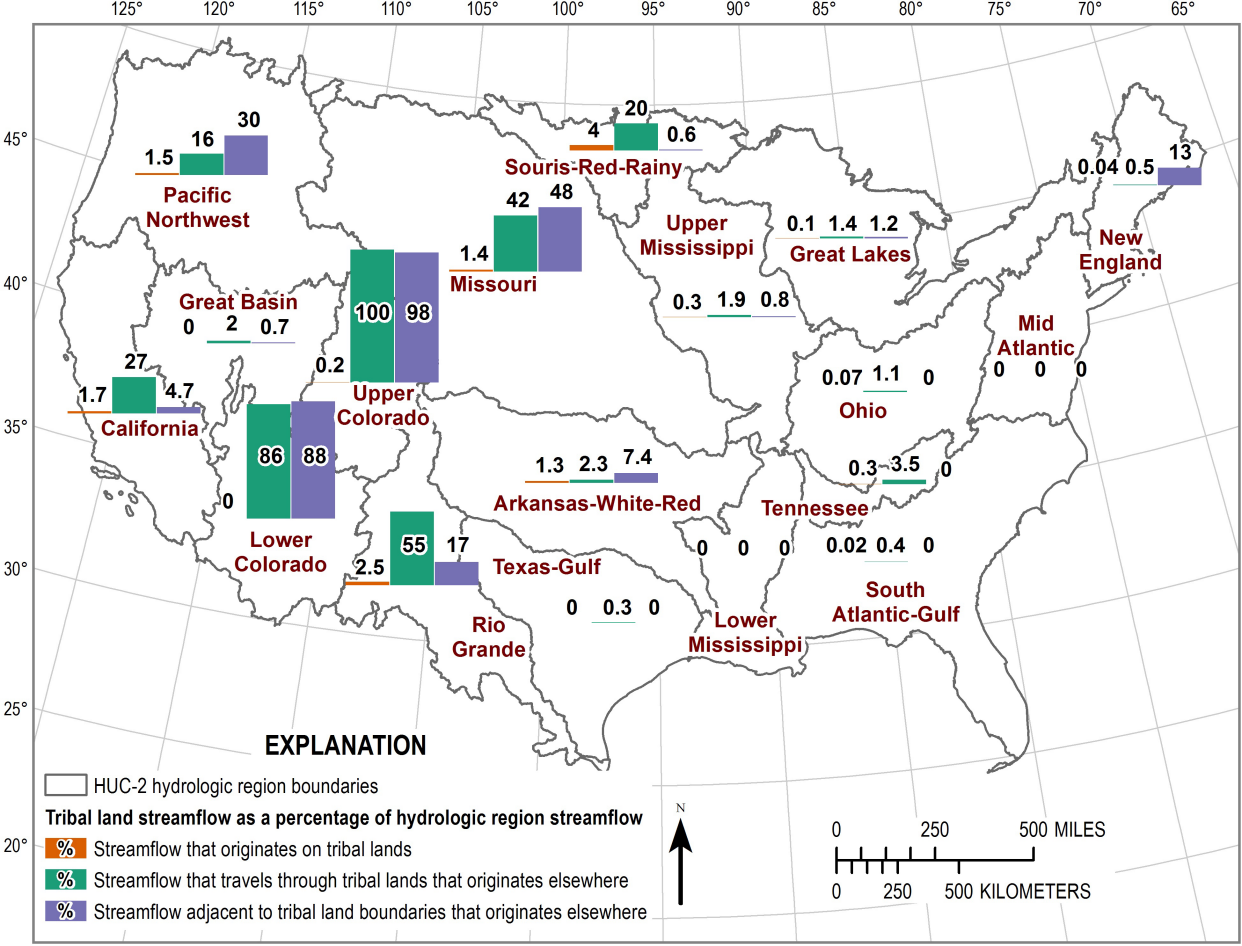
Tribal land streamflow that originates on tribal lands, travels through tribal lands, or runs adjacent to tribal as a percentage of 2-digit hydrologic unit code region streamflow.

There are larger quantities of streamflow that flow through or run adjacent to tribal lands than streamflow originating on tribal lands (Fig 5). Total streamflow exiting tribal lands and adjacent to tribal lands accounts for 9.53 percent and 11.32 percent, respectively, of total streamflow exiting the conterminous US. Approximately 115 percent of streamflow in the Upper Colorado region flows out of tribal lands even though most of the streamflow does not originate on those lands. The value is greater than 100 percent because of diversions and other losses in streamflow from the main channel. Similarly, over 86 percent of flows in the Lower Colorado region flow out of or adjacent to tribal lands. Tribal lands are associated with more than about 10 percent of streamflow in many HUC2 regions in the western US when combining streamflow through or adjacent to tribal lands (Table 2).

## Discussion

The magnitude of the national streamflow estimate for tribal lands of approximately 0.43 percent is consistent the other estimated water budget components. Given that the area of tribal lands is 3.36 percent, precipitation is 1.87 percent, and excess precipitation is 0.95 percent of values for the conterminous US, it is likely that tribal lands streamflow contributions are lower than these percentages. The accuracy of the water budget estimates depends on the accuracy of the data in the national datasets, which in turn are primarily based on generalized algorithms and limited measurements. The EROM estimates of streamflow were calculated using streamflow gaging stations with a period of record 20 years or greater [34]. Additional streamflow gages could be added to improve EROM streamflow estimates by easing the requirement of a 20-year record [34]. National AET datasets developed using remote sensing data may offer improved AET and water use estimates for HUC2 regions and tribal lands [40]. Full water balances for the HUC2 regions and tribal lands should include groundwater inflows, groundwater outflows, changes in groundwater and reservoir storage, and interbasin water transfers to improve the accuracy of streamflow origination estimates. Additionally, the integration of continental-scale model output could provide alternative climate data for the 1971–2000 period as well as more recent years.

Use of national datasets to estimate water budget components by HUC2 regions introduced inherent limitations to the analysis. First, the data used were for the period 1971–2000 and may not be representative of prior or successive time periods. Changes in climate over the past decades and into the future will likely alter the water budget components presented here. Generally, if temperatures across the western US increase over the next decades, then AET will increase and reduce excess precipitation and streamflow. For example, within the tribal lands of the Columbia River basin climate change has been identified as the cause of streamflow declines [41]. Of particular concern in this basin is the median reduction of April-July flow volumes by 16 percent from 1900 to 2009, as calculated from information collected by the USGS Hydro-Climate Data Network [41]. Second, polygon resolution and accuracy were a major determinant in identifying water budget values and streamflow locations. Discrepancies in tribal boundary polygons were particularly noticeable where the boundary was known to be coincident with a particular watershed boundary or stream, but differed from NHDplus datasets. NHDplus data were manually included for these cases to maintain the integrity of the tribal boundary polygons. Third, non-tribal inholdings on tribal lands were considered part of the tribal lands and were not removed from the water budget calculations. Discrete tribal lands that are less than about 10 square kilometers in size were preserved in the area, precipitation, AET, and excess precipitation accounting, but were not included in streamflow calculations as their contributions were considered negligible. Fourth, the national water use dataset was available at the HUC8 level, which in some cases blends tribal lands with nearby areas that have very different land and water use characteristics.

The use of national datasets provides numerical estimates of the contributions of tribal lands and tribal waters to the major US river basins and a comparative guide to the contribution of tribal water resources in different regions. It is necessary to state that these datasets are not sufficiently accurate to calculate water budgets for any single tribal land or reservation for local water resource management; a more accurate accounting of tribal water resources at the local level is warranted for these purposes [42–44].

The streamflow estimates for tribal lands presented in this paper are conservative as they are limited to federally-recognized political boundaries of tribal lands (as of 2017). The streamflow estimates would be higher if the analysis also included the waters and lands identified through treaty rights to support fishing, hunting, gathering, and habitat protection. These treaty rights could substantially increase the contribution of tribal lands as well as what could be more broadly defined as tribal waters for basins in the Pacific Northwest [45–47]. After passage of the McCarran Amendment, 43 U.S.C. & 666, in 1952, jurisdiction over tribal water use has become increasingly complex with the interplay of treaty based reserved water rights and state based water rights, and accordingly varies from state to state [48, 49]. As such, jurisdiction over tribal water use is beyond the scope of this paper. Additionally, the analysis in this paper is limited to the conterminous US and does not include lands with aboriginal interests in Alaska, Hawaii, and the Territories of the US.

## Conclusions

This analysis used national datasets representing precipitation, evapotranspiration, excess precipitation, streamflow, and water use for the period 1971–2000 to estimate the proportion of these hydrologic budget components that are associated with tribal lands. While tribal lands account for only 3.4 percent of the conterminous US and about 0.43 percent of streamflow origination, approximately 9.5 and 11.3 percent of US streamflow flows through tribal lands or adjacent to tribal land boundaries, respectively. In western HUC2 regions these numbers increase substantially. Between 86 and 100 percent of streamflow travels through or adjacent to tribal lands within the Upper and Lower Colorado HUC2 regions, respectively. For these regions, nearly every management decision impacting streamflow could influence water traveling through tribal lands. Resource management decisions occurring upstream of tribal lands will have an impact on these reservations, and resource management decisions on tribal lands will accordingly impact downstream users. Management of water resources within and external to tribal land boundaries may necessitate collaboration to meet respective goals such as preservation of Native American cultures, sustainable economies, healthy ecosystems, and access to clean drinking water. This is especially true for watersheds and aquifers shared by tribal and non-tribal entities experiencing significant land-use change, climate change, natural disaster response/resiliency, or the installation and operation of water resources infrastructure. This analysis is intended to inform these management strategies as they pertain to tribal lands at the national and regional levels.
